# Advance in placenta drug delivery: concern for placenta-originated disease therapy

**DOI:** 10.1080/10717544.2023.2184315

**Published:** 2023-03-08

**Authors:** Miao Tang, Xiao Zhang, Weidong Fei, Yu Xin, Meng Zhang, Yao Yao, Yunchun Zhao, Caihong Zheng, Dongli Sun

**Affiliations:** a College of Pharmaceutical Science, Zhejiang University of Technology, Hangzhou, 310014, China; b Department of Pharmacy, Women’s Hospital, Zhejiang University School of Medicine, Hangzhou, 310006, China

**Keywords:** Placenta, nanoplatforms, drug delivery, retention effect, pregnancy

## Abstract

In the therapy of placenta-originated diseases during pregnancy, the main challenges are fetal exposure to drugs, which can pass through the placenta and cause safety concerns for fetal development. The design of placenta-resident drug delivery system is an advantageous method to minimize fetal exposure as well as reduce adverse maternal off-target effects. By utilizing the placenta as a biological barrier, the placenta-resident nanodrugs could be trapped in the local placenta to concentrate on the treatment of this abnormal originated tissue. Therefore, the success of such systems largely depends on the placental retention capacity. This paper expounds on the transport mechanism of nanodrugs in the placenta, analyzes the factors that affect the placental retention of nanodrugs, and summarizes the advantages and concerns of current nanoplatforms in the treatment of placenta-originated diseases. In general, this review aims to provide a theoretical basis for the construction of placenta-resident drug delivery systems, which will potentially enable safe and efficient clinical treatment for placenta-originated diseases in the future.

## 1. Introduction

Over 130 million infants are born globally every year (Keelan et al., 2015), during whose pregnancies more than 20% (>26 million per year) suffered from one or more pregnancy-related complications. The most common disorders are preeclampsia, fetal growth restriction, gestational diabetes, and preterm birth. Among the numerous pregnancy complications, placenta-originated pregnancy complications, such as preeclampsia and fetal growth restriction, arising from abnormal placental development and function, are the most difficult diseases to treat in obstetrics for the lack of safe and effective drugs (Tang et al., 2022). Timely delivery of the baby is the most effective strategy to treat preeclampsia or fetal growth restriction. However, about 81% of the newborns survived from early-onset fetal growth restriction (before 32 weeks), wherein 12% of the surviving babies are diagnosed with cognitive impairment and/or cerebral palsy (Pels et al., 2020). Every year, about 70,000 pregnant women and 500,000 fetuses or newborns die of preeclampsia worldwide (Rana et al., 2019). Moreover, placenta-originated disorders also increase the risks of cardiovascular and metabolic diseases of mothers and babies in the long term, potentially increasing serious health concerns.

Deep knowledge of the placental function in transferring drugs and nutrients from the mother to the fetus will help researchers and clinicians make measures to improve maternal and fetal health (Al-Enazy et al., 2017). The placenta develops rapidly as a well-organized and functioning organ in early pregnancy to support fetal growth (Figueroa-Espada et al., 2020). In early pregnancy prior to the gestation of 10–12 weeks, the placenta is not completely developed to transfer nutrients proficiently (Figueroa-Espada et al., 2020; Koren & Ornoy, 2018). From the 10–12 weeks of gestation, the developing placenta has grown to supply nutrients, exchange waste products, and protect the fetus from exposure to some xenobiotics and toxic substances in the maternal circulation (Faber et al., 1992; Gude et al., 2004). From the aspect of maternal-fetal drug delivery, the placenta can be regarded as a crossing passage, flexibly and intendedly delivering therapeutics to treat pregnancy-related complications (Grafmueller et al., 2013; Joshi, 2017). Placental drug transfer depends on the physiological and pathological characteristics of the placenta and the physicochemical properties of drugs (Figueroa-Espada et al., 2020; Tuzelkox et al., 1995). The placenta has a high hemodynamic characteristic, which provides a guarantee for the delivery of nutrients from the mother to the fetus, and the various transporters in the placenta also provide opportunities for forward and reverse transport (Tetro et al., 2018). The physical and chemical properties of drugs themselves, such as drug molecular size, hydrophilic or lipophilic characteristics, also play an important role in their placental transportation. The placental transfer ability is affected by the molecular size of the therapeutic agent, which might be relatively impermeable when its molecular weight is greater than 1000 Da, and permeable when its molecular weight is less than 600 Da (Tuzelkox et al., 1995). Moreover, according to the like-dissolves-like principle, hydrophobic drugs can pass through the placental barrier more easily especially when they have a lower rate of protein binds and less ionization (Audus, 1999; Unadkat et al., 2004). In contrast, hydrophilic molecules are less permeable and cannot easily penetrate blood vessel walls and placenta. However, the most common forms of the drugs are chemical molecules with small sizes, which possess the possible procedures of easily crossing through the placenta (Syme et al., 2004), possibly causing severe fetal toxicity, such as abortion (Suarez et al., 2001), birth defects (Kulaga et al., 2011), and carcinogenicity (Venn et al., 2004) for the drug use during the pregnancy. For instance, a Dutch clinical trial of sildenafil for fetal growth restriction showed that sildenafil was associated with fetal pulmonary hypertension and fetal death (Hawkes, 2018). The most challenging problem of treating placenta-originated diseases is to take both efficacy and safety into therapeutic design considerations (Joshi, 2017).

**Figure 1.**
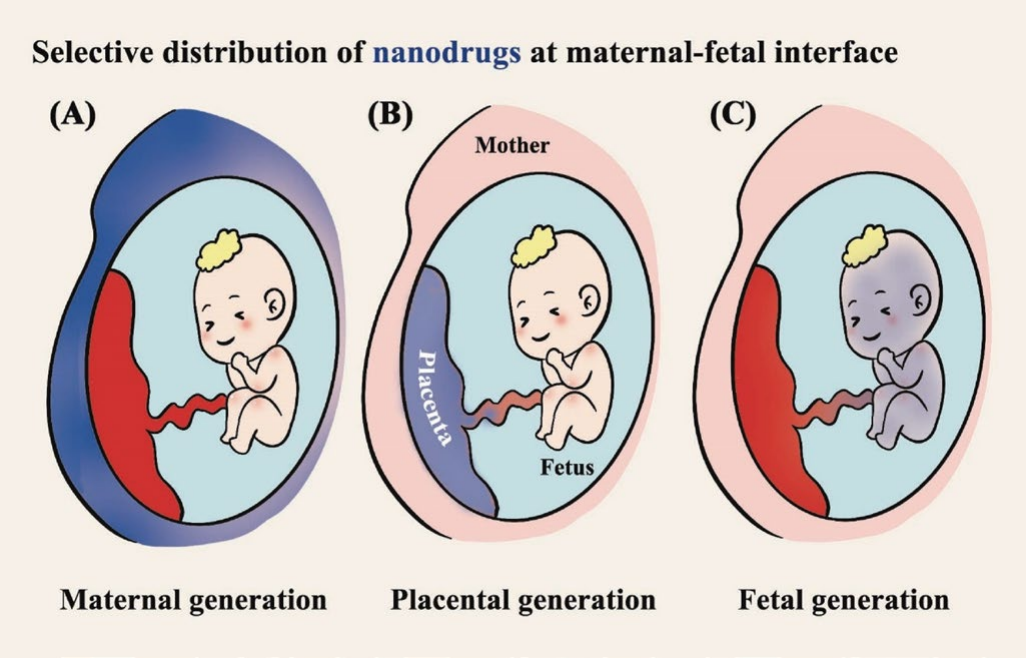
Application strategies for nanoparticle-mediated drug delivery in pregnancy. Three therapeutic scenarios are depicted: (A) treatment of maternal conditions without fetoplacental exposure; (B) treatment of placental conditions without maternal or fetal exposure; (C) treatment of fetal conditions without maternal or placental exposure.

Compared to small molecule drugs, the design of nanoplatforms could target the lesions and lower unnecessary therapeutics delivered as the off-target effects. Recently, many researchers have reported that advanced nanotechnology would assist in the treatment of pregnancy complications with safety and efficiency (Table 1) (Nelson et al., 2021; Pritchard et al., 2021). The placental permeability of nanoparticles could be designed via the adjustment of their physicochemical properties. Meanwhile, well-designed nanoplatforms may vary in distribution according to the therapeutic condition to be confined in the maternal side, easily pass through the placenta and enter the fetal circulation, or retain in the placenta (Pritchard et al., 2021). For example, one study showed that targeting nanoplatforms loaded with doxorubicin could deliver drugs to placental tissue. It can improve the therapeutic effect of ectopic pregnancy and reduce systemic toxicity (Kaitu’u-Lino et al., 2013). Alternatively, for placenta-originated diseases such as preeclampsia and fetal growth restriction, they could be designed to retain in the placental surface, having functions on the syncytiotrophoblasts (SCT) where the pathogenic factors produce and cause the development of the disease (Figure 1). In general, nanotechnology is able to keep the efficacy and reduce the side effect during the therapy of placenta-originated disease.

**Table 1.** Representative studies exploring the application of nanotechnology for placenta-originated disease therapy.

Disease	Nanocarriers	Administration methods	Therapeutic action	Model	Outcome	Ref.
Fetal growth restriction	Liposome	Not applicable	Increase EGR expression	Human placental explants	Increased nutrient transport capacity of placental explants	(Renshall et al., 2021)
	Liposome	Tail vein injection	Increase IGF2 expression	The P0 mouse model of fetal growth restriction	Increased placental weight and improved fetal weight	(King et al., 2016)
	Polyplex	Intra-placental injection	Increase IGF1 expression	Placental insufficiency mouse model	Increased the labyrinth thickness and pup weight	(Abd Ellah et al., 2015)
	Polymer	Tail vein injection	Antioxidation	Prenatal hypoxia rat model	Prevent oxidative stress in placentas	(Aljunaidy et al., 2018)
	Polymer	Not applicable	Increase hIGF1 expression	*In vitro* syncytiotrophoblast models	Functional changes to cellular activity and protection for oxidative stress	(Wilson et al., 2020)
Preeclampsia	Dendrimer siRNA complex	Tail vein injection	Inhibit sFlt-1 expression	Pre-eclamptic rat model	Increased embryonic weight while reducing hypertension, proteinuria, and sFlt-1 level	(Yu et al., 2017)
	Lipid-polymer nanoparticle	Intravenous injection	Down-regulating Nrf2 and sFlt1	Adult pregnancy-associated hypertension mice	Alleviated symptoms of preeclampsia	(Li et al., 2020a)
	Lipid-polymer nanoparticle	Intravenous injection	Decrease sFlt-1 expression	Pregnant CD1 mice	Decreased the protein and mRNA expression of sFlt-1 in serum	(Li et al., 2020b)
	Cholesterol siRNA complex	Tail vein or subcutaneous injection	Inhibit sFlt-1 expression	Pregnant CD1 mice and a baboon preeclampsia model	Decreased the mRNA expression of sFlt-1 in mouse placenta and no change in pup weight	(Turanov et al., 2018)

Nanotechnology has been clinically used to treat tumors and can overcome off-target effects and achieve targeted drug delivery. However, due to the high research cost, long research cycle, and safety issues, the application of nanotechnology in placenta-originated diseases is delayed (Tetro et al., 2018). Now, advances in the understanding of placental features have made it possible to realize the application of nanotechnology in placenta-related disease therapy. To provide a theoretical basis for the construction of placenta-resident drug delivery systems for placenta-originated disease therapy, this review summarizes the mechanism of nanoplatform transport in the placenta, and analyzes the physicochemical properties (including particle size, charge, and surface modification) of nanocarriers and pregnancy stages that affecting the placental retention of nanoplatforms. More importantly, we analyzed the advantages and attention issues of nanoplatforms in the treatment of placenta-originated diseases. In general, this review will provide guidelines for the construction of placenta-resident drug delivery systems and bring new hope for the therapy of placenta-originated diseases.

## 2. Placental barrier for the specific design of the nanodrugs

**Figure 2.**
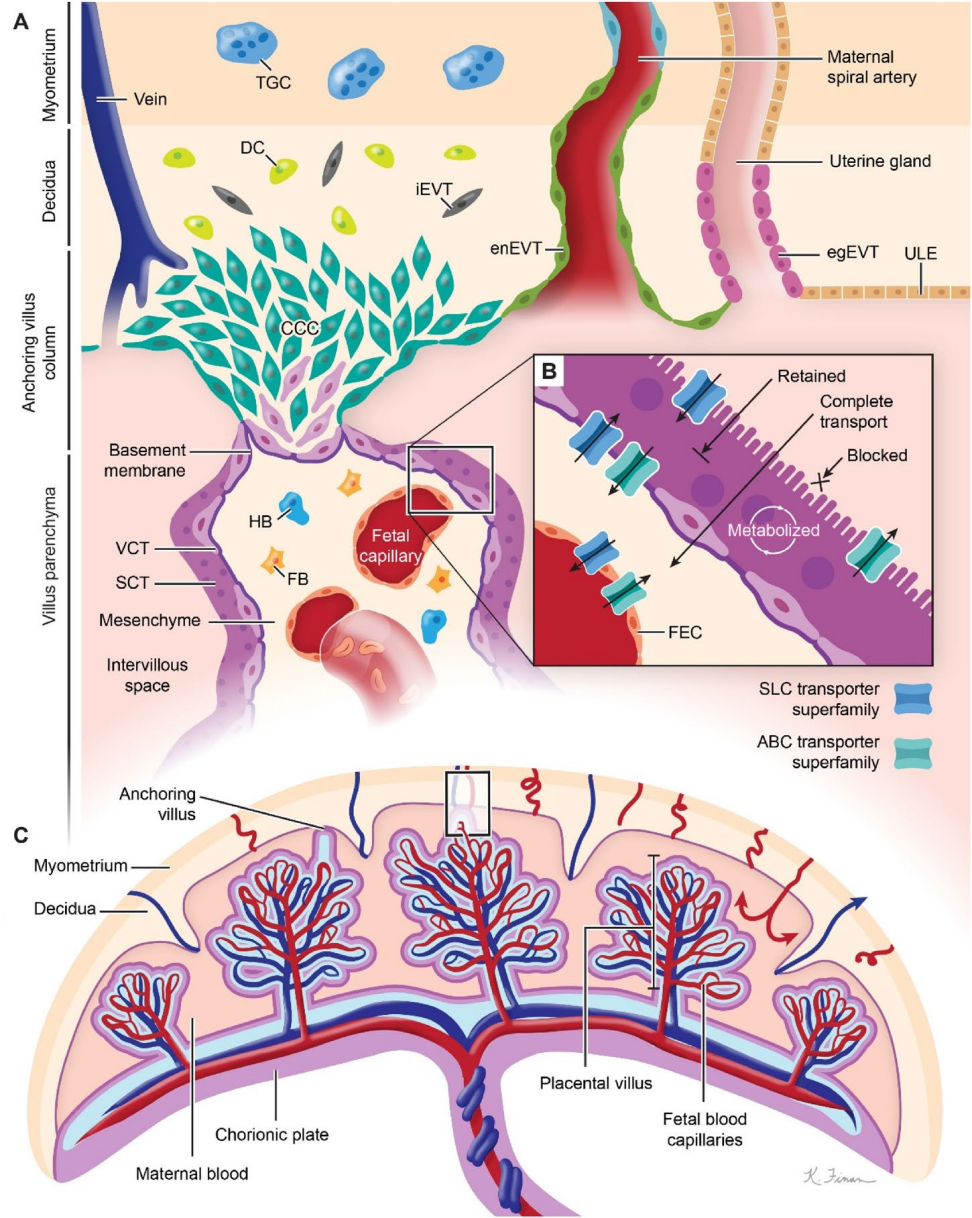
Diagram of the physiological structure of the placenta. (A) Modified spiral arteries enable sufficient perfusion of the placenta with maternal blood that bathes the intervillous space and makes direct contact with the SCT. (B) SCT is a master regulator of placental translocation. (C) Fetal blood enters the placenta via the umbilical artery (blue) and flows into the capillaries in the placental villi before returning to the fetus via the umbilical vein (red). (SCT: syncytiotrophoblasts, VCT: villous cytotrophoblast, TGC: trophoblast giant cells, HB: Hofbauer cells, FB: fibroblasts, CCC: cytotrophoblast cell column, DC: decidual cells, iEVT: interstitial extravillous trophoblast, enEVT: endovascular extravillous trophoblast, egEVT: endoglandular extravillous trophoblast, ULE: uterine luminal epithelium, FEC: fetal endothelial cell). Image from reference (Arumugasaamy et al., 2020) cited with permission. Copyright © 2020 Elsevier B.V.

The human placenta is a discoid monochorionic double-perfused organ (Figure 2C). The placenta develops primarily from the trophectoderm surrounding the blastocyst. With the progress of embryo implantation, trophectoderm cells gradually differentiate and develop into a variety of trophoblast cell subtypes with specialized functions. These cell types include villous cytotrophoblasts (VCT), syncytiotrophoblasts (SCT), extravillous trophoblasts (EVT), and trophoblast giant cells. In addition to trophoblasts, other cells that make up the placental environment include Hofbauer cells, fibroblasts, fetal endothelial cells, and decidual cells (Arumugasaamy et al., 2020). During pregnancies, maternal vasculature undergoes structural changes to allow efficient blood flow to the fetus (Figure 2A). Uterine arteries develop various branches; basal arteries end in the decidua or myometrium; and spiral arteries extend to the intervillous space (Brosens et al., 2019). When VCT arrive in the endometrium, their growth and proliferation become faster. External VCT cells fuze to develop multinucleated SCT that can produce enzymes that promote extracellular matrix (ECM) degradation and secrete factors that induce the apoptosis of endometrial epithelial cells for blastocyst implantation (Gupta et al., 2016). VCT with proliferative ability can grow toward the maternal decidua to form the cytotrophoblast cell column (CCC), and be anchored in the maternal decidua. VCT then differentiate into invasive extravillous trophoblasts (EVT) at the ends of CCC. Invasive EVT develop into interstitial extravillous trophoblasts (iEVT) or into intravascular extravillous trophoblasts (enEVT) (Ji et al., 2013; Roberts et al., 2017; Velicky et al., 2016). Eventually, iEVT enter the inner third of the myometrium as multinucleated giant cells, along with enEVT, altering arteries to ensure efficient blood flow and adequate nutrient supply (Burton et al., 2019).

Special physiological conditions during pregnancy help nanodrugs to be trapped in the placenta (Valero et al., 2018). For one thing, the blood flow of the uterine artery increases sharply during pregnancy, and the resistance of flow in the blood vessel is reduced due to the distal segment expansion of the spiral artery. For another, due to the hemodynamic adjustment, maternal blood circulation volume and cardiac output increase (Burton et al., 2009). When nanodrugs were administered intravenously, high placental blood flow increased the transportation of the nanodrugs into the placenta. The nanodrugs reach the placental intervillous space through the spiral artery. The blood flow velocity in the intervillous space decreases rapidly to 10 times lower than that in the uterine artery, prolonging the contact time between the nanodrugs and the chorionic villi, as well as the SCT (Burton et al., 2009). The outer side of the apical membrane of the SCT is a brush-like structure with a large surface area, increasing the possibilities of nanodrugs internalization and local drug release (Arumugasaamy et al., 2020). Furthermore, transport proteins in the apical membrane, such as ATP-binding cassette (ABC) and solute carrier protein (SLC) transport proteins, might be utilized as efficient tools for the delivery or efflux of nanodrugs (Figure 2B) (Arumugasaamy et al., 2020). Overall, the nanodrugs may take advantages of the placental structure, the placental hemodynamics as well as the transporters for placenta-originated disease therapy.

## 3. Transport mechanism of nanodrugs in the placenta

The key to treating placental diseases is to concentrate the drug into the placenta as much as possible to minimize the side effects to the fetus and mother. It is important to understand the delivery mechanism of nanoplatforms in the placenta before constructing a placenta-resident drug delivery system. In the placenta, nanoparticles can be transported by common transcellular transport mechanisms such as passive diffusion, active transport, and pinocytosis. The exact transport pathway may depend on the physicochemical properties of nanoplatforms (Al-Enazy et al., 2017; Shojaei et al., 2021). The following section will introduce the mechanism of nanoplatforms entering and exiting the placenta. More importantly, some strategies to enhance the accumulation of nanoplatforms in the placenta were summarized to provide new ideas for researchers to treat placenta-originated diseases.

### 3.1. Paracellular pathway

**Figure 3.**
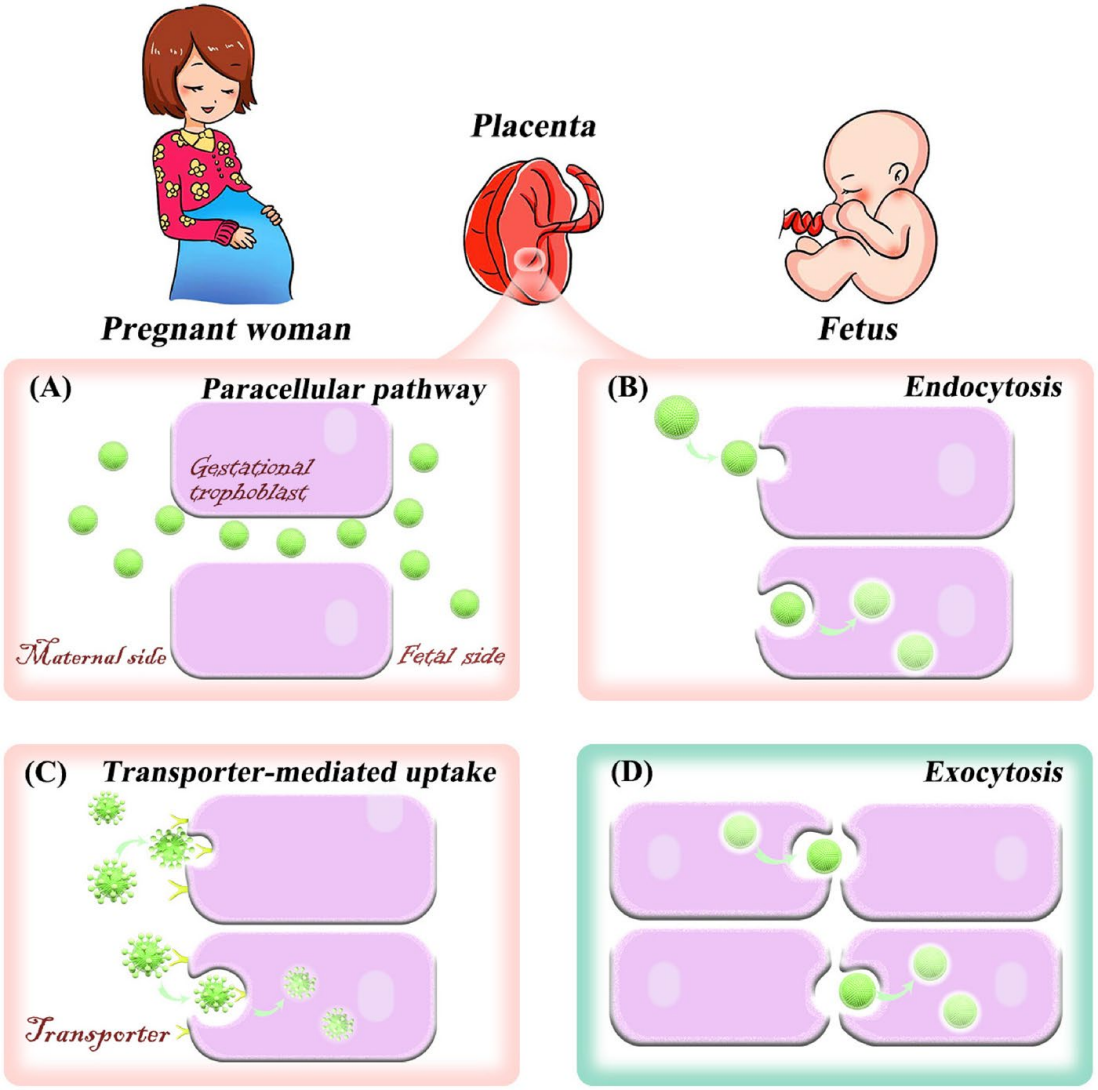
Transport of nanodrugs in the placenta. Trophoblast uptake through the paracellular pathway (A), endocytosis (B), and transporter-mediated pathway (C). Trophoblast exocytosis of nanoplatforms (D).

Paracellular pathway is a process by which substances are absorbed through the intercellular space. It is a passive mode of transport that consumes no energy. Studies reported that the placenta has various lipid pores (Kertschanska et al., 1997; Kurz & Fasching, 1968). Subsequently, Kertschanska et al. found that such placental pores extended from the basal trophoblast surface to the SCT. Alternatively, by using electron microscopic analysis, Kertschanska et al. reported that the lipid pore has approximately a diameter of 15 to 25 nm at normal intravascular pressure (Kertschanska et al., 1997). In addition, several studies have reported that the placental pores (channels) are continuous and curved from the fetus to the mother (Bosco et al., 2007; Kertschanska et al., 2000; Kurz & Fasching, 1968). Small hydrophilic compounds such as opioid peptides (Ampasavate et al., 2002) and nanoparticles smaller than 25 nm in diameter will be allowed to pass through the placenta by passive diffusion due to the presence of placental channels. The entry of the nanoplatforms through the pores into the placenta is known as paracellular absorption (Figure 3A). An *in vivo* study has shown that the injection of quantum dots smaller than 25 nm into pregnant mice was more likely to pass through the placenta than those larger ones, and the transferred number of quantum dots increased with the dose (Chu et al., 2010). In addition, small dendritic nanoparticles (5.6 nm) can cross the placenta via placental channels (Menjoge et al., 2011). These studies indicate that nanoplatforms with particle sizes less than 25 nm can enter the placenta through the paracellular pathway, but they can also easily cross the placenta through continuous placental channels. Therefore, the nanoplatforms with small particle sizes may not have a good placental resident effect, and may even affect fetal development. Notably, the development of stimuli-responsive aggregated nanoplatforms provides a new way to overcome this obstacle (Mura et al., 2013; Yu et al., 2020; Zhang et al., 2020). The main idea of this strategy is that small particle-size nanoparticles can specifically aggregate into large particle sizes in the placental environment, which can not only improve placenta-specific aggregation and long-term retention but also enhance the treatment effect of placental diseases. This strategy has been applied in the study of tumor-targeted drug delivery. A study showed that acid-responsive aggregated gold nanoparticles can specifically accumulate and retain in the acidic tumor microenvironment (Luan et al., 2022). The placenta and tumor are similar to some extent (King et al., 2016). Thus, such a strategy could be applied in the design of placenta drug delivery systems.

### 3.2. Endocytosis

Endocytosis is the process of transporting extracellular substances into the cell through the deformation movement of the plasma membrane, which is divided into phagocytosis and pinocytosis. Pinocytosis is the main pathway of nanoparticle uptake and can be classified into two categories: clathrin-mediated endocytosis and clathrin-independent endocytosis (Conner & Schmid, 2003; Zhang et al., 2019). Clathrin-independent endocytosis pathway includes macro endocytosis and caveolae-mediated endocytosis. There are many clathrin-coated regions in the trophoblast of the placental syncytium between microvilli (Zhang et al., 2019). Thus, nanoparticles can be absorbed by the trophoblast through pinocytosis (Figure 3B). Studies have shown that polymer nanoparticles with positive charges, as well as gold nanoparticles, can be internalized by SCT through clathrin-mediated endocytosis and caveolae-mediated endocytosis (Kaul et al., 2013; Myllynen et al., 2008; Rattanapinyopituk et al., 2014). In a 2018 study, pullulan acetate nanoparticles were internalized into BeWo B30 placental barrier cells via caveolae-mediated endocytosis (Tang et al., 2018). Rattanapinyopituk et al. in 2014 investigated clathrin- and caveolin-mediated transport of gold nanoparticles in the placenta by intravenous injection (Rattanapinyopituk et al., 2014). The results showed that gold nanoparticles increased the expression of caveolin in fetal endothelial cells, as well as the clathrin in SCT and fetal endothelial cells. In the study, gold nanoparticles could pass through the placenta and enter fetal circulation through clathrin- and caveolin-mediated endocytosis (Rattanapinyopituk et al., 2014).

These results suggest that both clathrin- and caveolin-mediated cellular uptakes may be explored for placental targeting drug delivery. Based on this transport mechanism, researchers can modify clathrin or caveolin easily recognized molecules on the surface of nanoplatforms to achieve the purpose of placental delivery of drugs.

### 3.3. Transporter-mediated uptake

Transporter-mediated uptake is divided into facilitated diffusion and active transport. Facilitated diffusion allows certain compounds to cross the placenta without energy. Active transport is an energy-dependent process that usually proceeds against a concentration gradient. The major superfamily of transporters found in the placenta are the SLC and ABC transporters (Al-Enazy et al., 2017; Staud et al., 2012). For instance, organic anion transporters are a family of transporters in the placenta, mediating transport in the maternal-fetal interface for metabolites, waste products, and hormones (Lofthouse et al., 2018). Similarly, transporters such as amino acid transporters, glucose transporters, and transferrin can deliver specific substrates across the placenta (Illsley, 2000; Parkkila et al., 1997). For example, iron is transported across the placenta through transferrin receptor-mediated endocytosis (Parkkila et al., 1997).

Facilitated diffusion transport increases the transport rate of endogenous compounds, such as hormones and nucleosides, when passive diffusion cannot meet the functional and metabolic needs of the fetus. Transport of drugs via facilitated diffusion pathways appears to be rare (Syme et al., 2004), and there have not been any reports of nanoparticle-facilitated diffusion. Meanwhile, active transport mechanisms for placenta-specific substrate delivery have not been well studied for their utilization in drug delivery. At present, the active transport of nanomaterials through this mechanism is mainly focused on the field of cancer therapy. In one study, stealth liposomal systems modified with aspartate-polyoxyethylene stearate conjugate (APS) were designed to target ATB^0,+^ overexpressed human lung cells via transporter-mediated delivery (Luo et al., 2017). However, with the development of nanoscience, known placental transport mechanisms can be exploited for receptor-mediated drug delivery with high specificity into the placenta (Figure 3C). Our group has previously explored the transporters that highly expressed in the human placenta (Zeng et al., 2019), and confirmed that nucleoside transporters could mediate the entry of their substrate-modified liposomes into gestational trophoblasts (Fei et al., 2021). In conclusion, researchers can increase placental drug delivery by constructing nanoplatforms modified with high-affinity substrates for transporters in the placenta.

### 3.4. Exocytosis

Exocytosis is the process that transport vesicles release their contents into the extracellular matrix through fusion with the plasma membrane. After being ingested by cells, nanoparticles will undergo a series of pathways in the cell and eventually be transported out of the cell (Dahiya & Ganguli, 2019; Sakhtianchi et al., 2013). The transport of nanoparticles across placental tissue is mainly through exocytosis and can occur in two main ways (Figure 3D): (i) after endocytosis, nanoparticles are internalized into early endosomes. Early endosomes become mature and form into multivesicular bodies, then fuse with the plasma membrane and release nanoparticles from the trophoblast. Therefore, nanoparticles reach fetal circulation; (ii) early endosomes transport nanoparticles to lysosomes, and then exocytosis of lysosomes can also release the contents into the villus matrix and subsequently into fetal capillaries. From the mechanism of nanoplatform transferring out of the placenta, it can be seen that the construction of nanoplatforms that can achieve lysosomal escape can make nanoplatforms stay in the placental trophoblast cells for a longer time and reduce the amount of placental transmission.

This section describes the mechanism of nanoplatform transport in the placenta. More importantly, based on these transport mechanisms, this review has summarized some strategies for the construction of drug delivery systems that are expected to improve the placental residence. These strategies are summarized in three aspects: the first aspect is to increase the affinity of the nanoplatforms with placental trophoblast cells, that is, to modify the substrate of cell membrane transporters on the surface of nanoplatforms; the second aspect is to achieve nanoparticle aggregation through the placental microenvironment and increase placental retention; finally, lysosomal escape of nanoplatforms could reduce the nanodrug leaving the placenta. It should be emphasized that these strategies are still in the theoretical stage, and follow-up studies are needed to confirm their feasibility.

## 4. Key factors affecting placental retention of nanoplatforms

The permeability of the placenta to nanoparticles with different physicochemical properties varies widely. The nanoparticles are devised according to the therapeutic purpose to control their distribution on the maternal, placenta, and fetal sides. Nanoparticles can be designed to easily penetrate the placenta and participate in fetal circulation, or they can be designed to prevent drugs through the placenta and remain in the maternal compartment to maximize maternal drug concentrations while minimizing injurious effects on the fetus, or they can be designed to more precisely target the placenta and accumulate in its superficial layer, SCT, etc. for the treatment of placenta-originated diseases. The barrier imposed by the placenta to nanodrug transport is strongly affected by the physicochemical properties of the nanoparticles, including particle size, surface charge, nanomaterial type, and surface modification. Additionally, one must take into account how placental physiology and transport vary at different stages of gestation while designing nanoplatforms. After comprehensive research, it is easier to develop a satisfactory placenta-resident drug delivery system, enhancing the targeting effect and improving the bioavailability of drugs.

### 4.1. Particle size

**Figure 4.**
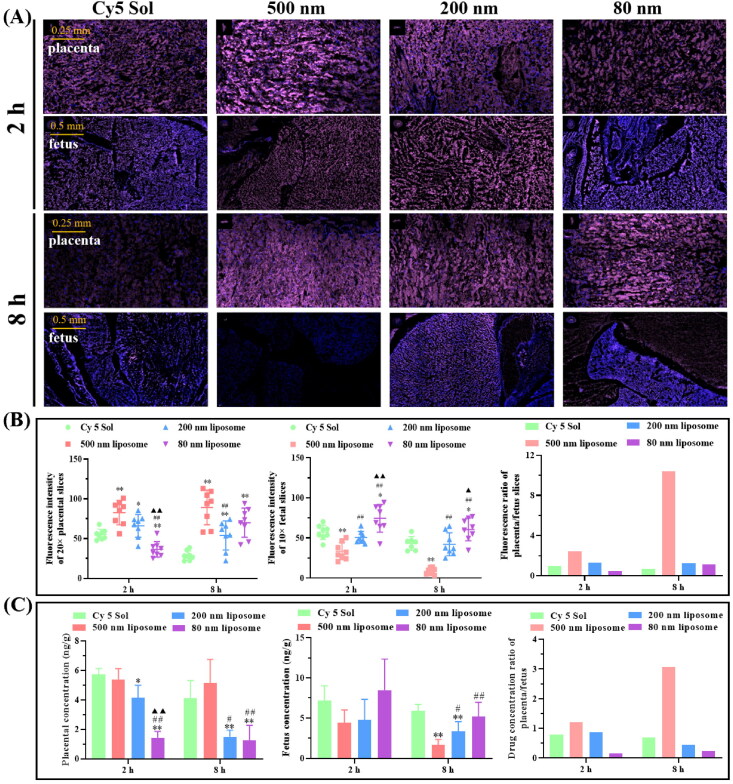
Pregnant mice injected intravenously with Cy 5 solution or Cy 5-loaded liposomes of different sizes were processed and analyzed after 2 and 8 h. (A) Fluorescent sections containing Cy 5 in the placenta and fetus of pregnant mice. (B) Cy 5 fluorescence intensity in placenta and fetus of pregnant mice. (C) Quantitative analysis of Cy 5 content in the placenta and fetal tissues of pregnant mice by liquid chromatography-mass spectrometry. Image from reference (Tang et al., 2022) cited with permission. Copyright © 2022 Elsevier B.V.

The size is a major factor influencing the retention of nanoparticles in the placenta, and typically, the placental barrier is less restrictive for smaller-sized lipophilic nanoparticles. Therefore, nanoplatforms of larger size and hydrophilicity may be more suitable for the treatment of maternal or placenta-originated diseases to avoid drug transfer from the placenta to the fetus. Our research group prepared 80 nm, 200 nm, and 500 nm Cy 5-loaded liposomes and then studied their aggregation in the placenta (Tang et al., 2022). The results of fluorescence intensity and liquid chromatography-mass spectrometry analysis of placenta and fetal slices showed that the particle size of liposomes was positively correlated with the accumulation of liposomes in the placenta (Figure 4A–C). After 8 hours of administration, the Cy 5 concentration ratio of placenta to fetus was analyzed, and the ratio of 500 nm lipids was about 6 times higher than that of the 200 and 80 nm-sized liposomes, indicating that 500 nm liposomes were more suitable for placental drug delivery (Figure 4C) (Tang et al., 2022). Ali et al. found that the permeability of dexamethasone-loaded PLGA nanoparticles was halved when the size of PLGA nanoparticles was increased from 143 to 196 nm in an *in vitro* model of human placental cells (Ali et al., 2013). In another study, Refuerzo et al. tested whether silicone nanovectors (SNVs) at 519 nm, 834 nm, and 1000 nm would cross the placenta in pregnant rats (Refuerzo et al., 2011). They demonstrated that 1000 nm SNVs did not cross the placenta and remained in the maternal circulation, while smaller SNPs (close to 500 nm) could cross the placenta and participate in the fetal circulation. Huang et al. investigated whether fluorescently labeled carboxylate-modified polystyrene nanoparticles at 20, 40, 100, 200, and 500 nm could cross the placenta and affect trophectoderm function, ultimately finding that only 40 nm nanoparticles had prominent placenta ingestion and trophectoderm internalization (Huang et al., 2015).

In general, the particle size of the nanoparticle will affect its placental retention in the placenta. When constructing the nanocarrier, selecting an appropriate particle size is necessary for placenta drug delivery, thus improving the safety and reliability of nanoplatforms for the treatment of pregnancy-related diseases.

### 4.2. Charge

Surface charge is another property that determines whether nanoparticles will transfer from the placenta to the fetus. Cationic nanoparticles are more likely to cross the placenta than anionic nanoparticles because of the easier uptake of cationic nanoparticles by the negatively charged membrane of trophoblast cells (Zhang et al., 2019). In an *in vitro* blood-placental barrier model, Müller et al. verified that cation-coated superparamagnetic iron oxide nanoparticles (SPIONs) had the strongest ability to interact with BeWo cells and were predominantly retained in the BeWo/pericytic layer. In comparison, anionic and neutral surface-charged SPIONs could cross the cell layer more readily (Müller et al., 2018). Ho et al. demonstrated the effect of surface charge by simultaneously injecting anionic or cationic multimodal polymer nanoparticles in the first and third trimesters of pregnant rats, respectively. In the third trimester, cationic nanoparticles aggregated more easily than anionic nanoparticles in the chorionic plate of the rat placenta, while in the first trimester, both nanoparticles readily penetrated the placenta. The above results emphasized that electrical charge and different gestational stages affected the placental uptake of nanoparticles (Ho et al., 2017).

It should be noted that positively charged nanoparticles are removed from the bloodstream faster than negatively charged nanoparticles due to increased tissue and cellular uptake, and they can induce hemolysis and platelet aggregation (Albanese et al., 2012; Nel et al., 2009). Di Bona et al. reported that cationic nanoparticles accumulated preferentially in the mouse placenta at high doses, but also increased the risk of maternal and fetal toxicity (Di Bona et al., 2014). Therefore, investigators must carefully control the surface charge of nanoplatforms to balance tissue toxicity and placental transport.

### 4.3. Different materials

Different materials of nanoparticles have different permeability extents in the placenta. For example, gold nanoparticles above 80 nm, silica nanoparticles above 300 nm, and polystyrene nanoparticles above 500 nm all remained in the placenta without entering the fetus (Aengenheister et al., 2021; Bongaerts et al., 2020). Even if the nanoparticles are of similar size, different materials produce different placental resident effects. In an *ex vivo* perfusion model, 4–8 nm TiO_2_ NPs were unable to pass through the placenta (Aengenheister et al., 2019), whereas 3–6 nm Au NPs were able to cross the human placental barrier and enter the fetal circulation (Aengenheister et al., 2018). Under the same particle size, organic nanomaterials can enter or cross the placenta more easily than inorganic nanoparticles, because organic nanoparticles always have deformability (elasticity) and they are more likely to enter the placenta through paracellular pathways. For instance, many studies reported that both 50–500 nm liposomes and 20–500 nm polystyrene nanoparticles could enter the placenta and accumulate in the fetus (Irvin-Choy et al., 2020). In comparison, 80 nm gold nanoparticles and silica nanoparticles over 300 nm aggregated very little in the placenta, and they were not detectable in the fetus. From this point of view, organic nanoparticles have broader particle size selectivity in placental drug delivery. Furthermore, vesicle-like nanocarriers such as liposomes and exosomes can also enter the placental barrier through membrane fusion. This type of nanocarrier is often used for drug delivery in placenta-originated diseases due to its superior placental aggregation ability (Tang et al., 2022). Notably, few inorganic nanocarriers have been used for drug delivery in placenta-originated diseases (Table 1). This is because inorganic carriers, such as silicon dioxide, titanium dioxide, etc., degrade slowly in the body and easily cause embryotoxicity. This is an even more important issue (safety of nanocarriers) than drug delivery, which would be discussed in the following pages.

### 4.4. Surface functionalization influences placental aggregation or transmission of nanoplatforms

The surface functionalization of nanoparticles has many advantages, such as reducing toxicity and immune response, increasing the specificity and efficacy of the nanodrugs. For instance, ligands modified on the surface of the nanoparticles could increase the specificity and efficacy of the nanodrugs. Meanwhile, the surface modification could also help to change the efflux and penetration of nanoplatforms. The molecules used to modify the nanoparticles include small proteins, peptides, antibodies, aptamers, and oligosaccharides (Sanita et al., 2020).

#### 4.4.1. Modification of placental targeting groups increases placental aggregation

To better target the placenta, nanoplatforms can be surface modified by targeting ligands, such as peptides, antibodies, or aptamers that bind placenta-specific receptors (Whigham et al., 2019). The mechanism of placental targeted nanodrugs is that the ligands on the surface of the nanoplatforms bind specifically to receptors on the surface of trophoblast cells or other placental cells. This bond is supported by intermolecular forces such as van der Waals forces, hydrogen bonds, etc. Such interacting forces allow the nanoparticles to stay on the surface or inside the target cells. Therefore, the nanoplatforms modified by targeting groups can show a good placental residence effect. Tumor-homing peptides, such as CGKRK and iRGD, whose receptors are expressed at high levels in the human placenta, have developed into feasible targets for placenta-specific drug delivery (Beards et al., 2017; King et al., 2016). When A. King et al. injected T7 phage displaying surface peptides CGKRK or iRGD intravenously into pregnant mice, the enrichment of CGKRK and iRGD was approximately 7- to 8-fold higher in the placenta compared with other organs. They observed that CGKRK- and iRGD-modified nanoparticles bound to murine decidual spiral arteries and the vasculature of the placental labyrinth, but not to the junctional zone adjacent to the labyrinth, at different gestation periods. Meanwhile, in the human placental explant model, CGKRK- and iRGD-modified nanoparticles could accumulate rapidly in the outer syncytium rather than permeating into the underlying cytotrophoblast. Subsequently, they used iRGD-modified targeted liposomes to selectively deliver insulin-like growth factor 2 to the mouse placenta, improving fetal body weight distribution in growth-restricted model mice. Tumor-homing peptides provide new ideas for developing placenta-specific therapies (King et al., 2016). The two placenta-targeting peptides were inspired by the similarity between tumors and the placenta. In the future, we can use the similarity between tumors and the placenta to develop more placenta-targeting nanodrugs (Table 2).

**Table 2.** Characteristics of nanoplatforms that would be beneficial in treating pregnancy complications.

Placenta-targeting molecules	Receptor	DDS	Targeting effect/retention effect	Outcomes	Ref.
CGKRK	Membrane-associated calreticulin	Liposomes	The enrichment of CGKRK in the placenta was increased by about 8 times compared with other organs.	Tumor-homing peptides facilitate targeted delivery of peptide-decorated liposomes to the mouse placenta	(King et al., 2016)
iRGD (CRGDKGPDC)	α_ν_ Integrin	Liposomes	The enrichment of iRGD in the placenta was increased by about 7 times compared with other organs	Tumor-homing peptides facilitate targeted delivery of peptide-decorated liposomes to the mouse placenta	(King et al., 2016)
Synthetic placental CSA binding peptide	Placental chondroitin sulfate A	Nanoparticles	The accumulation of T-NP_Cy5-siRNA_ in the placenta was about 3 times as much as that in the unmodified group	T-NP_sisFLT1_ selectively accumulate in placenta and downregulate sFlt-1 expression in mice	(Li et al., 2020b)
Synthetic peptide CNKGLRNK	Endothelium of the uterine spiral arteries and placental labyrinth	Liposomes	Targeted preparations could still be observed in the placental labyrinth and decidual spiral artery 48 hours after administration	CNKGLRNK-decorated liposomes selectively bound to the endothelium of the uterine spiral arteries and placental labyrinth *in vivo*	(Cureton et al., 2017)
OTR-antibody	OTR	Immunoliposomes	The fluorescence intensity of immunoliposomes in the uterus was 7 times that of untargeted liposomes.	Immunoliposomes efficiently delivered indomethacin to the uterus and reduced the rates of preterm birth compared with untargeted liposomes	(Paul et al., 2017)
EGFR antibody	EGFR	Nanocells	Only the targeted preparation group showed strong doxorubicin fluorescence in syncytiotrophoblast cells at 7 hours after administration	EGFR-targeted EnGeneIC Delivery Vehicles loaded with doxorubicin significantly inhibited trophoblastic tumor cell growth	(Kaitu’u-Lino et al., 2013)
CSA-binding peptide	Placental chondroitin sulfate A	Lipid-polymer nanoparticle	Only the targeted treatment group detected the drug in the placenta 48 hours after administration	plCSA-BP facilitate targeted delivery of nanoparticles decorated with plCSA-BP to the placental trophoblasts	(Zhang et al., 2018b)
CSA-binding peptide	Placental chondroitin sulfate A	Lipid-polymer nanoparticle	Targeted nanoparticles could be absorbed by JEG3 cells within 30 minutes	plCSA-BP facilitate targeted delivery of nanoparticles decorated with plCSA-BP to the placental trophoblasts	(Zhang et al., 2018d)
CSA-binding peptide	Placental chondroitin sulfate A	Lipid-polymer nanoparticle	The amount of CSA targeted preparation absorbed by JEG3 cells was 3 times that of the nontargeted group	Targeted delivery of DOX to choriocarcinoma	(Zhang et al., 2018a)
CSA-binding peptide	Placental chondroitin sulfate A	Lipid-polymer nanoparticle	Only the targeted treatment group detected the drug in the placenta 48 hours after administration	Targeted delivery of methotrexate to the placental trophoblasts	(Zhang et al., 2018c)

**Figure 5.**
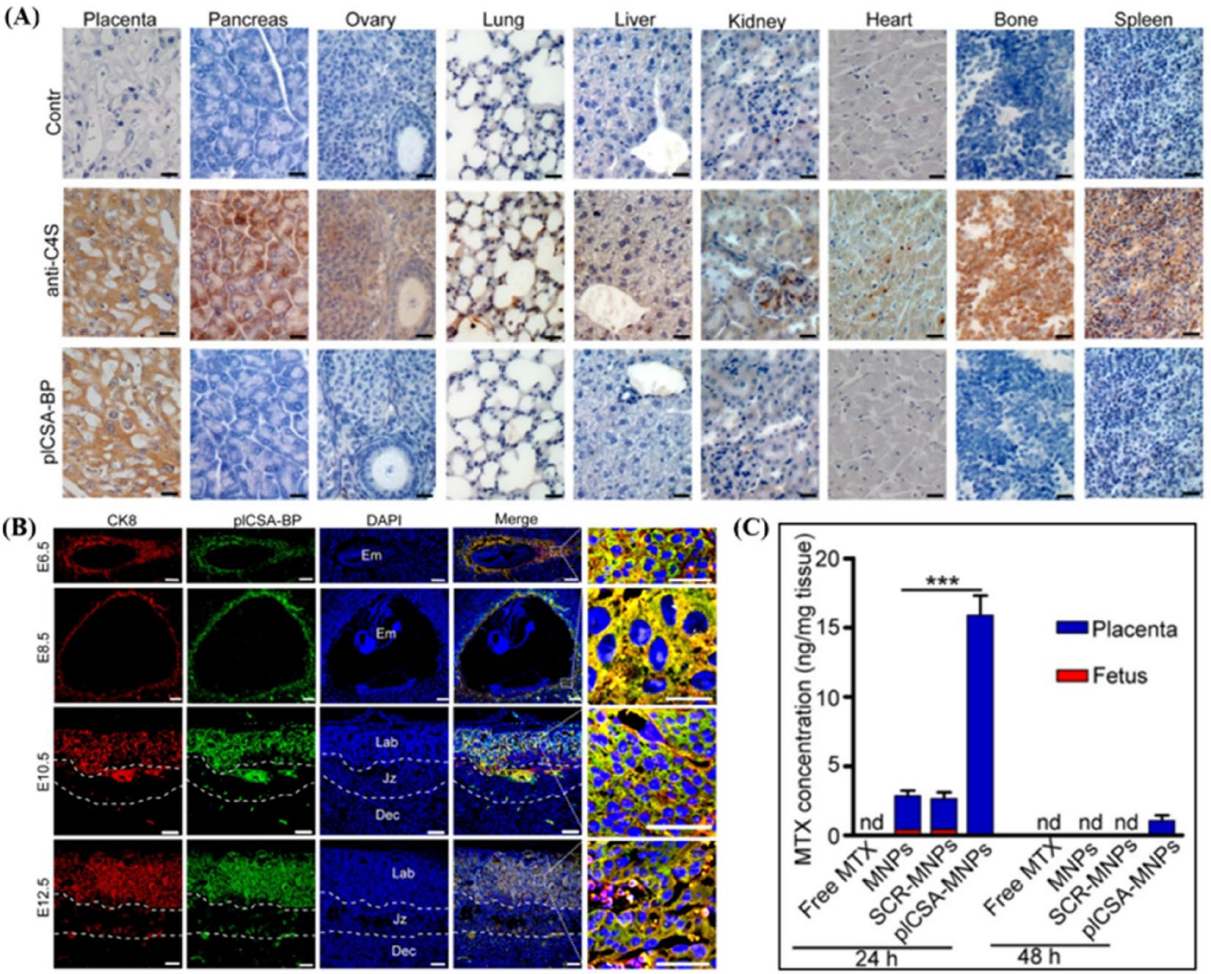
(A) Analysis of sections of different tissue blocks incubated with anti-C4S (2B6) and plCSA-BP revealed that plCSA-BP specifically binds to placental tissue. (B) plCSA-BP specifically binds to trophoblast at different gestational stages in mice. Trophoblast cells (CK8, red), biotin-plCSA-BP (green), and nuclei (DAPI, blue). Dec: decidua; Em: embryo; Jz: junctional zone; Lab: labyrinth. (C) Quantitative analysis of MTX concentrations in placenta and fetuses by HPLC. nd: not detected. MNPs: Lipid-polymer nanoparticles loaded with MTX; SCR-MNPs: The scrambled peptide-conjugated nanoparticles loaded with MTX; plCSA-MNPs: plCSA-BP-conjugated nanoparticles loaded with MTX. Image from reference (Zhang et al., 2018b) cited with permission. Copyright © Ivyspring International Publisher.

Chondroitin sulfate A (CSA) is present in the placental SCT, and placental CSA-binding peptide (plCSA-BP) can specifically bind to CSA in the trophoblast. Zhang et al. showed that plCSA-BP conjugated nanoparticles efficiently bound to mouse placental maze trophoblast cells *in vivo* and *ex vivo* (Figure 5A,B). plCSA-BP conjugated nanoparticles were used as delivery vehicles for methotrexate (MTX). In the targeted therapy group, the MTX concentration in the placenta was 6-fold higher than that in the non-targeted therapy group at 24 hours, while MTX was undetected in the fetus. After 48 hours of dosing, MTX was detected only in the placenta of the targeted therapy group (Figure 5C). It shows that plCSA-BP conjugated nanoparticles can increase the accumulation of drugs in the placenta and prolong the action time of drugs (Zhang et al., 2018b). CNKGLRNK is a novel peptide sequence that selectively binds to the uteroplacental vascular system. CNKGLRNK peptide-modified liposomes could not enter fetal circulation and directly treated endothelial cells in the uterine spiral artery and placental labyrinth (Cureton et al., 2017).

Another study found that three fluorescein-labeled elastin-like peptides (ELPs) ranging from 25 to 86 kDa (4.1 to 6.8 nm) were unable to cross the placental barrier when administered intravenously. The most prominent accumulation location of nanoparticles is the placental chorionic plate, and the accumulating concentration of nanoparticles increased with sizes (Kuna et al., 2018). ELPs are genetically encoded, which means that researchers have absolute control over the ELP sequence and molecular weight, and targeting peptides and therapeutic proteins can be easily added by modifying the DNA-encoding sequence. ELPs are biologically inert macromolecules, and ELP fusions can stabilize the protein, peptide, or small molecule cargoes in the body’s circulation. Tunability, long circulation, biodegradability, and non-immunogenicity make them ideal nanocarriers for drug delivery during pregnancy, delivering drugs to the placenta while preventing fetal drug exposure.

Recently, researchers have identified exosomes as attractive candidate therapeutic agents and delivery nanoplatforms (Lu & Huang, 2020). Exosomes are lipid bilayer nanovesicles with different sizes (30 to 150 nm). The bioactive entities packaged in exosomes can be transferred between cells, resulting in changes in recipient cell phenotype (Vader et al., 2016). Compared with artificial nanoparticles, exosomes have multiple advantages as drug delivery vehicles: lower immunogenicity and toxicity, direct fusion with target cell membranes, stronger cellular internalization, fewer off-target effects, etc. (Zhang et al., 2019). Placental cells can communicate with maternal tissues through exosomes to regulate their biological functions, and exosomes are present in higher numbers in pregnancy complications such as preeclampsia and gestational diabetes (Salomon & Rice, 2017). The surface of exosomes derived from different tissues contains protein fragments that bind to the ligands of the tissue cells. Such ligand-receptor interaction mechanism can realize the specific aggregation of exosomes (Liang et al., 2021). Placenta-originated exosomes can target the trophoblast to deliver the payload directly to the placenta. Meanwhile, the exosomes can be modified with functional ligands to improve the stability of blood circulation, better localize to the target site, and increase the efficiency of intracellular delivery. In general, placenta-originated exosomes may be a targeted vehicle for the treatment of placenta-originated diseases.

#### 4.4.2. Surface-functionalized nanoparticles reduce placental penetration

Surface-functionalized nanoparticles can promote placenta-specific drug delivery and reduce nanoparticle transfer to the fetus, thereby improving drug safety and efficacy. Polyethylene glycol (PEG)-coated liposomes were nearly impermeable to the placental barrier (Shojaei et al., 2021; Soininen et al., 2015). In an *ex vivo* placental perfusion model, Myllynen et al. demonstrated that PEG-coated 10–30 nm gold nanoparticles (Au NPs) were not able to penetrate the human placenta within 6 h of perfusion (Myllynen et al., 2008). PEG acted as a hydrophilic shell, repelling the adsorption of opsonins and other serum proteins to the nanoplatforms, and preventing macrophage clearance with ‘stealth’ properties (Suk et al., 2016). In another study, three surface modifications, namely ferritin with good biocompatibility, PEG with stealth effect, and stabilizing anionic material citrate, were used to decorate 13 nm Au NPs to evaluate the influence of surface function on the biodistribution of Au NPs in the maternal-fetal interface (Yang et al., 2012). Ferritin-modified or PEGylated Au NPs had similar levels of placental accumulation, in contrast to the significantly reduced fetal tissue uptake of citrate-terminated Au NPs. This suggests that the addition of stealth agents (e.g. PEG) to nanoparticles inhibits their ability to cross the placenta. However, it is worth noting that excess PEG also inhibits uptake by parent tissues and cells, thus requiring a balance between PEG surface modification, placental transport, and maternal cellular uptake.

PEG is the most commonly used surface-modifying molecule for the pharmaceutical formulation, thus, existing studies have focused on the effect of PEG surface modification on the placental aggregation of nanodrugs. With the deepening of research, the effects of other commonly used surface-modifying compounds (Guerrini et al., 2018), such as zwitterionic ligands, lipid bilayer, and proteins, on the placental generation of nanodrugs should also be investigated. Overall, this aspect is still in its infancy, and more efforts need to be devoted to the development of nanoplatforms with placental aggregation capabilities.

We have just mentioned some important key factors affecting the placental retention of nanoplatforms, including particle size, charge, material type, and surface modification. However, other factors such as the morphology of the nanoplatforms and physical conditions such as temperature and pH of the particles are also important, but information on their mechanisms affecting placental accumulation and penetration is lacking. More research is needed to gain a deeper understanding of these factors affecting nanoparticle placental retention. In addition, chemical structure modification of drugs and regulating the expression of transporters in placental cells are effective strategies to reduce placental drug efflux.

### 4.5. Pregnancy stage

In addition to the physicochemical properties of nanoplatforms, the gestational age of pregnancy is another key factor affecting the placental retention of nanoplatforms. The structure, cellular composition, function, and blood flow of the placenta vary with gestational age, thereby altering placental uptake and transport capacity. During early pregnancy, when the placenta is not yet filled with maternal blood, most drugs in the maternal blood enter the embryo by passive diffusion. By the end of the first trimester, the placental barrier can be as thick as 20 microns, and the placental retention ability is strong. The placenta thins to 2 to 5 microns at term, which means the fetus is more likely to be exposed to maternal drugs at this time. In addition, placental blood flow and surface area increase significantly with gestation time, which also enhances the likelihood of drug transfer to the fetus (Pritchard et al., 2021).

Yang et al. evaluated the influence of gestational age and nanoparticle composition on placental transfer by maternal administration of nanoparticles in mice (Yang et al., 2012). Experiments showed that gold nanoparticles injected intravenously in early gestation (< E11.5) in mice had higher fetal accumulation compared to late gestation (E > 11.5). Pietroiusti et al. intravenously injected SiO_2_ nanoparticles of three different sizes (25, 60, and 115 nm) and two different surface functionalizations (NH_2_ and COOH) at three different gestational time points in pregnant mice (Pietroiusti et al., 2018). The results showed that only 60 nm negatively charged nanoparticles could produce fetal accumulation at all three stages, while larger nanoparticles could only cross the placenta in the third trimester. At mid-pregnancy, 25 nm nanoparticles were the only ones of the three nanoparticles that could pass through the placenta in both positively and negatively charged forms. The ratio of the concentration of negatively charged SiO_2_ nanoparticles in the placenta to that in the fetus was highest in the third trimester of pregnancy. That is, the accumulation of 115 nm SiO_2_ nanoparticles in the placenta was twice that of the fetus, and 60 nm SiO_2_ nanoparticles was three times higher. What’s more, 25 nm negatively charged SiO_2_ nanoparticles were only detected in the placenta (Pietroiusti et al., 2018).

The *in vivo* transport process of drug delivery systems is complex and will affect the placental drug delivery efficiency. For instance, the reticuloendothelial system and plasma albumin complexation will accelerate the clearance of nanodrugs and reduce drug accumulation in the placenta. The drug released by nanodrugs at the placenta may also return to the maternal side or enter the fetal side through diffusion. That meant the placental resident effect of nanodrugs is also related to the loaded drugs. From the discussion in this section, constructing placenta-resident nanoplatforms is challenging. Given that the development of each field is gradual, we call for more attention and research to focus on the field of placental drug delivery to improve the maternal-fetal safety of drugs.

## 5. Potential superiority of nanoplatforms in placenta-originated disease therapy

### 5.1. General advantages of nanodrugs

Different types of nanocarriers have been successfully adopted in various nanodrugs, such as liposomes, albumin, and polymer nanoparticles. Because nanocarriers have unique properties, such as various shapes, sizes, and physicochemical properties that give them a high surface area to volume ratio, and the ability to carry therapeutically active compounds that eventually aggregate at the target site (Edis et al., 2021). When nanoparticles focus on placental drug delivery, their unique targeting properties combined with various strategies could distinguish them from traditional treatment during pregnancy. First and foremost, nanoparticles have flexible cargo encapsulation, widening their delivery of drug categories such as small molecule compounds, peptides, proteins, RNA, and DNA. Their large surface area and volume ratio also enable high loading capacity for drugs via different intermolecular forces. Therefore, nanoparticles can be designed by various construction options, suiting different pharmaceutical applications and cargo properties. Drug cargo would avoid degradation or metabolism with the help of protective encapsulation, keeping therapeutically active at the targeted site. Second, nanoparticles can penetrate the placental barrier flexibly via alterations of size, charge, and shape, enhancing on-site drug concentrations for disease therapy. Third, the versatile surface structure or surface charge of nanoparticles can control immunogenicity, inflammatory potential, and clearance. One example is the ‘stealth’ technology, where PEG is attached to the surface of nanoparticles. This surface modification can reduce the uptake by the mononuclear phagocyte system (MPS) to prolong the presence of PEGylated nanocarriers in the blood (Immordino et al., 2006). Finally, nanoparticles could be designed to improve placental drug delivery by different therapeutic mechanisms, such as active targeting through surface modifications and passive targeting by the placental microenvironment.

### 5.2. Improve placental retention and reduce maternal-fetal drug exposure

The significant physiological changes that occur during pregnancy have created an urgent need to develop drug delivery technologies specifically for use during pregnancy, such as pregnancy-induced increases in circulating blood volume and cardiac output by nearly 50%. Pharmacokinetics, including blood clearance and biodistribution, were different compared to non-pregnant women (Gude et al., 2004; Tasnif et al., 2016). Most treatments used clinically are not tissue-specific, then the drug accumulates in maternal and fetal tissues, leading to off-target toxicity (Refuerzo et al., 2017). Therapeutic drugs are transported to the target site or organ in a controllable manner by drug delivery systems, maximizing the therapeutic effect while minimizing the off-target effects of the administered therapeutic agents. Thus, nanoparticles can be used to precisely control drug delivery during pregnancy and reduce the risk of fetal and maternal drug exposure.

The placentas have many characteristics in common with solid tumors, such as rapid proliferation, avoidance of immune destruction, induction of angiogenesis, etc. (Lala et al., 2021). Zhang et. al compared placenta-specific methotrexate delivery with general methotrexate delivery by conjugating a placental targeted peptide or a scrambled peptide respectively to the nanoparticles. After intravenous administration of these nanoparticles to pregnant mice, they measured the cross-sectional areas of blood sinusoids in the placental labyrinthine region. It was reported that both targeted and non-targeted delivery of nanoparticles significantly decreased the mean blood sinusoid areas in the placenta, indicating that nanoparticles might take advantage of the enhanced permeability and retention (EPR) effect to improve the delivery of drugs to the placenta (Zhang et al., 2018b). Therefore, it might be reasonable to conclude that the abundant new blood vessels in the placental tissue make the placenta have a certain resident effect on the nanoparticles, making liposomes accumulate in the placental tissue similar to the tumor EPR effect.

The drug delivery technology then enhances the placental retention of nanoparticles and reduces maternal-fetal drug exposure by manipulating the physicochemical characteristics of nanoparticles, including their size, surface charge, composition of the material, and surface modification, taking into account different stages of pregnancy (Figueroa-Espada et al., 2020). For example, hydrophobic and positive-charged particles can increase placental tissue uptake and reduce drug placental penetration compared to hydrophilic and uncharged particles (Keelan et al., 2015). Drugs can be attached to macromolecular carriers such as cyclodextrins (Andaluz et al., 2013), or placental penetration of drugs can be reduced by using colloidal drug delivery systems such as liposomes or dendrimers (Menjoge et al., 2011). Additionally, liposomes have been successfully used to encapsulate small molecular drugs such as valproic acid (Barzago et al., 1996), inulin (Tuzelkox et al., 1995), riboflavin (Tuzelkox et al., 1995), methotrexate (Tuzelkox et al., 1995), penicillin (Tuzelkox et al., 1995), and indomethacin (Refuerzo et al., 2015) to increase the accumulation of these drugs in the placenta. Nanocarriers with surface-modified conjugation, such as targeting peptides, accumulate in target organs, thereby maximizing drug delivery to the desired location (Whigham et al., 2019), reducing the risk of drugs interference with normal placental and/or fetal development, and ultimately increasing drug safety. Zhang et al. mixed siRNAs against nuclear factor-erythroid 2-like 2 (Nrf2) and Soluble fms-like tyrosine kinase 1 (sFlt-1) to construct nanoparticles and achieve the synchronous downregulation of Nrf2 and sFlt-1 in the placenta (Li et al., 2020a). The nanoparticles were constructed by the carboxyl-polyethylene glycol-poly (d,l-lactide) (COOH-PEG5K-PLA8K), cationic lipid 1,2-Dioleoyl-3-trimethylammonium propane (DOTAP), and a conjugating peptide. The surface modification by conjugating peptides targeting CSA enabled the nanoparticle to deliver drugs to the placenta accurately. The treatment effects and pregnancy outcomes in nanodrug-treated mice were significantly better than those observed with single gene inhibition.

### 5.3. Placental microenvironment-specific drug release

Stimuli-responsive nanocarriers have received a great deal of attention for their versatility. Nanocarriers are designed to modulate drug release at the target site by inducing their action through endogenous, including pH, temperature, enzymes, and oxidative reduction. For instance, the variation of glutathione concentration between the tumor microenvironment and normal tissues creates a platform for the generation of the redox-sensitive drug delivery system incorporated with disulfide bonds (Yang et al., 2022). Different pH between normal tissues and tumor microenvironment provides opportunities for pH-sensitive drug delivery systems (Sethuraman et al., 2021). Although the placenta microenvironment has not been connected to the design of nanodrugs, we may apply it as a reference for constructing placenta-specific drug release systems. It was reported that the extracellular microenvironment of the trophectoderm exhibits a lower pH due to hypoxia-induced lactate production (Kay et al., 2007). Hest et al. designed an ELP diblock copolymer that disassembled under mildly acidic conditions (Abdelghani et al., 2021).

## 6. Attention issues of nanoplatforms in placenta-originated disease therapy

### 6.1. Safety of nanocarriers

The safety of nanoparticles is paramount when considering the use of nanoparticles in reproductive medicine. The exposure of nanodrugs to unwanted tissue (maternal or fetal) must be reduced or eliminated, while still providing sufficient therapeutic benefit. Because of low maternal tolerance during pregnancy, any genetic or epigenetic changes in the fetus caused by nanoparticles in the uterus have the potential to cause long-term deleterious effects. The toxicity of nanomaterials is related to the chemical properties, transepithelial electrical resistance, particle size, surface modification, concentration, and paracellular permeability of nanomaterials (Ali & Rytting, 2014). The following are some examples of nanomaterials with maternal fetal toxicity and relatively safe nanomaterials.

Yamashita et al. found significant adverse effects of silica nanoparticles on the placental barrier, such as decreased blood flow, spiral artery injury, and apoptosis of cavernous trophoblast cells (Yamashita et al., 2011). Another study found genetic dysregulation in the fetal cerebral cortex, olfactory bulb, and areas associated with the dopamine system after fetal exposure to TiO_2_ NPs (Umezawa et al., 2012). Maternal lungs exhibited morphologically emphysema-like changes after intravenous injection of 30 nm diameter AuNPs in pregnant mice (Yang et al., 2014), whereas 100 nm AuNPs were genotoxic to fetal liver and blood and cause miRNA dysregulation in fetal lung and liver (Balansky et al., 2013). *In vivo* administration of 80 nm chromium and cobalt nanoparticles resulted in abnormal fetal hippocampal neurodevelopment and increased DNA damage (Hawkins et al., 2018). Ag accumulation in the mother might affect the growth of placenta and embryos, and induce epigenetic alterations in the embryos, contributing to postponing the cognitive and physical development of the fetus (Ema et al., 2017; Zhang et al., 2015). Long-term accumulation of quantum dots in the mother may increase the risk of embryonic dysplasia (Žalgevičienė et al., 2012).

High concentration of polystyrene nanoparticles decreased cell viability of choriocarcinoma cells (BeWo cell line) *in vitro* (Cartwright et al., 2012), which might be due to the positive correlation between high polystyrene dose and pro-inflammatory effects (Brown et al., 2001; Cartwright et al., 2012). Surface-modified polystyrene nanoparticles were not present in the fetus but accumulated in the liver, spleen or mesangium of pregnant mice with potential health risks (Kenesei et al., 2016). In the *in vitro* placental perfusion experiment, the placental transfer rate of polyamide dendrimers was significantly reduced compared with free drugs, and placental function was not affected (Menjoge et al., 2011). *In vitro* (BeWo cells and perfused placenta model), PEGylated doxorubicin liposomes exhibited lower placental penetrability than free doxorubicin and pH-sensitive liposomal preparations of doxorubicin, reducing fetal exposure (Soininen et al., 2015).

Risks to the mother and fetus from the various inorganic, organic, or hybrid nanoparticles described above vary, and some reviews also focused on the safety of nanoparticles (Das et al., 2016; Ema et al., 2016; Hou & Zhu, 2017; Keelan et al., 2015; Muoth et al., 2016; Zhang et al., 2017). Inorganic nanoparticles have shown the ease of crossing the blood-placental barrier and induce multiple toxicological effects (Pereira et al., 2020). The direct toxicity of inorganic and organic nanoparticles to fetus might be avoided by nanoparticle retention in the placenta, but the toxicity caused by the deposition of inorganic materials in the mother is still a problem (Verougstraete et al., 2018). In this aspect, biodegradable organic nanoparticles might be more advantageous, and their current clinical application as drug carriers further proves their safety (Anselmo & Mitragotri, 2016). However, both inorganic and organic nanoparticles have also been considered to have indirect toxicity, which may cause maternal pro-inflammatory effects and reproductive endocrine disruptors and indirectly affect the growth and development of the fetus (Gualtieri et al., 2014; Hutz et al., 2014). Therefore, this nascent area deserves more investigation. Compared with inorganic nanoparticles, organic nanoparticles have the potential for better targeting selectivity and less toxicological effects (Kannan & Kannan 2017; Pereira et al., 2020). In general, both inorganic and organic nanoparticles would be able to have good function in placenta drug delivery as long as researchers take advantage of their properties and design nanoparticles skillfully. Surface functionalization of nanoparticles can interfere with the transplacental passage and facilitate placenta-specific drug delivery to reduce maternal-fetal toxicity. Biodegradable materials show less direct toxicity than slowly metabolized biomaterials, but the indirect toxicity of the drug carriers still needs long-term concern. In addition, researchers can pay more attention to biologically derived substances and use their natural targeting, immune tolerance, and other characteristics to design safer vectors, such as placenta-originated exosomes. Besides the toxicity, the bioavailability, solubility, and stability issues of nanoplatforms also deserve more attention in placenta-originated disease therapy.

### 6.2. Placental penetration of nanoplatforms

Drugs always cross the placenta to a certain extent after maternal administration (Tetro et al., 2018). When researchers construct placenta-resident drug delivery systems, they need to pay attention to the placental penetration of nanodrugs, because the drug will pass through the decidua-placental junction and adversely affect the fetus during organogenesis and organ maturation. As one of the earliest drug carriers, liposomes have been widely studied for their placental transfer. For instance, T4 thyroxine is a kind of water-soluble macromolecule, whose penetration is effectively blocked by the placenta. After being encapsulated by liposomes, it could penetrate the placenta through the active transport pathway and improve the placental permeability of T4 thyroxine (Bajoria et al., 1997). In an *in vitro* model, poly(lactic-co-glycolic acid)-encapsulated dexamethasone increased dexamethasone transfer to the fetal compartment (Ali et al., 2013). Moreover, compared with free drugs, liposomal encapsulated penicillin, inulin, methotrexate, and riboflavin in the research were more localized to the placenta and more transferred to the fetus (Tuzelkox et al., 1995). These results demonstrated that nanoplatforms in some conditions could alter the ability of drugs to cross the placental barrier. The above discussion reminds researchers to carefully consider the compatibility of nanomaterials and small molecule drugs. Rapid development and metabolic changes during pregnancy make the fetus more sensitive to external toxicants. Therefore, when preparing nanoplatforms, we should not only consider the function of nanomaterials (placental retention and placental targeting), and the safety of nanomaterials (for both maternal and fetal safety), but also pay attention to the different transport and metabolism conditions of drugs encapsulated in nanoparticles. As the transport and metabolism of the modified drugs are closely related to efficacy and safety, we must clarify the placental penetration of nanodrugs, minimize the potential effect of the drug delivery system on the fetus, and prevent the occurrence of conditions that affect fetal development. The semi-quantitative or quantitative results on the accumulation of nanoplatforms in the placenta versus in the fetus should be included to evaluate their placenta-penetrating effect. Moreover, multiple analytical methods are needed to quantify and/or visualize the pregnancy transfer of nanoparticles, such as liquid chromatography-mass spectrometry analysis, magnetic resonance imaging, ultrasound imaging, photoacoustic imaging, etc. (Bongaerts et al., 2020).

### 6.3. Placental heterogeneity in humans and animals

A critical step in translating therapeutic and drug delivery technology to the clinic requires the use of animal models *in vivo* to assess therapeutic effects. The most commonly used animal model for preliminary preclinical studies is the mouse model. The mouse placenta shares a pivotal structural similarity with the human placenta in that both are choroidal placenta, which is characterized by direct contact between maternal blood and trophoblast tissue. Whereas there are several crucial differences between human and mouse placentas, as well as several pivotal differences in full reproduction, that must be taken into account when using mice as model organisms. Such as, mice have a particular inverted yolk sac or chorioflavin placenta. This critical difference can result in higher levels of toxicity to placental development in mice than that in humans when studying drug delivery techniques. In addition, the transport of therapeutic agents across the yolk sac placenta may differ from the human placenta and may have a stronger protective effect against certain foreign substances (Schmidt et al., 2015). The intraplacental anatomy is another important difference between the mouse placenta and the human placenta. The placenta of mice has junction areas and labyrinth areas, which are responsible for the endocrine function and maternal-fetal exchange respectively. In contrast, the human placenta contains two trophoblasts during early gestation, which evolve into a functional area containing lamellar trophoblasts with villi extending into the maternal blood lumen (Dilworth & Sibley, 2013). In conclusion, the mouse model does share similarities with the human placenta (the trophoblast and endothelium separate the maternal-fetal blood supply), but there are still significant differences in the structure, number of cell layers, and dispersion barrier thickness (larger in mice than in humans). Therefore, when assessing the potential, preclinical efficacy, and safety of transplacental delivery of nanoparticles, the results must make assumptions about the applicability to humans, and experimental studies can use a combination of *in vitro*, *ex vivo*, and *in vivo* models to make experimental results more reliable.

## 7. Conclusion and prospects

The Centers for Disease Control and Prevention reports that approximately 70% of pregnant women take at least one prescription drug and 90% of women overall take at least one medication while pregnant (Joshi, 2017). Over the past three decades, the use of prescription drugs during the first trimester of pregnancy has grown by more than 60%, the usage of four or more medications during the first trimester of pregnancy has almost tripled, and the use of four or more medications at any point throughout the pregnancy has more than doubled (Joshi, 2017). Nevertheless, the risk-averse pharmaceutical company is understandably hesitant to engage in the evaluation and development of treatments for pregnant women, haunted by the ghost of thalidomide and the possibility of disastrous lawsuits. This truth is plainly demonstrated by data that pregnant women are purposefully excluded from 98% of medication administration studies (Shields & Lyerly, 2013). Among all pregnancy-related disorders, the therapy of placenta-originated disorders like fetal growth restriction and preeclampsia is particularly difficult. Because it is very difficult to concentrate the drug in the placenta while minimizing the drug permeation on the fetal side. The majority of commonly used medications are small molecules that can pass through the placental barrier by passive diffusion, resulting in substantial fetal toxicity such as miscarriage, deformity, and carcinogenicity. As a result, therapeutic progress for placenta-originated illnesses has been modest in recent decades, and new technology and fresh techniques are required to ameliorate the situation.

Recent advances in nanoscience have made placenta-resident drug delivery systems a potential tool for the treatment of placenta-originated diseases, as the ability of nanodrugs to prevent drugs from crossing the placental barrier could greatly increase the number of drugs available to pregnant women. By adjusting the physicochemical properties of nanocarriers, the placenta-resident drug delivery system can optimize the speed and extent of drug transplacental transport and minimize unnecessary drug exposure to the fetus. To accelerate the achievement of constructing placenta-resident drug delivery systems, this review demonstrated the transport mechanism of nanodrugs in the placenta and analyzes the physical and chemical factors that affect the retention of nanoplatforms in the placenta.

By reviewing the literature, we have obtained some important insights: (1) the uptake ability of placental trophoblasts to different kinds of materials or nanocarriers with different physicochemical properties varies greatly. Therefore, researchers must screen ‘fetal-friendly’ nanodrugs for the effective treatment of placenta-originated diseases through a sufficient amount of preclinical research; (2) organic carriers are safer in the field of placental drug delivery, and have a relatively wide range of particle sizes choose space; (3) based on the similarity between tumor and placenta, an inspiration from the nanodrug of tumor treatment would help researchers to quickly step forward to achieve placenta drug generation in the treatment of placenta-related complications. At the same time, it should be noted that this similarity does not represent consistency. The knowledge of placental microenvironment and pathophysiology should be deeply studied and differences should be detected, in order to explore the truly suitable materials; (4) as mentioned above, some small molecules may become easier to pass through the placenta and lead to fetal exposure due to the design of nanoplatforms. Therefore, we need to further consider the compatibility between carriers and drugs, as the distribution and metabolism of the nanoparticles are also important for efficacy and safety. More importantly, the advantages and concerns of nanoplatforms in the treatment of placenta-originated diseases are summarized for guiding researchers to construct more potential nanotherapeutics for placenta-originated disease therapy.

The goal of placenta-originated disease therapy will take decades to become a clinical reality, and nanodrugs for maternal-fetal health are still in the early phases of development. Much effort is expected to optimize the various portions of the nanotherapeutics and bring the promise to realization. The biodistribution of nanotherapeutics during pregnancy will become more controlled or predictable as scientists continue to identify the ‘rules’ for the placental uptake and transport of nanodrugs. As a result, innovative technologies for treating pregnancy issues should thrive. We anticipate that the first nanotechnology-based pregnancy-specific medicinal formulations will be evaluated in the near future, with a view to developing new treatments for pregnant women and their fetuses.
